# Using the random forest method to detect a response shift in the quality of life of multiple sclerosis patients: a cohort study

**DOI:** 10.1186/1471-2288-13-20

**Published:** 2013-02-15

**Authors:** Mohamed Boucekine, Anderson Loundou, Karine Baumstarck, Patricia Minaya-Flores, Jean Pelletier, Badih Ghattas, Pascal Auquier

**Affiliations:** 1 EA3279, Self-perceived Health Assessment Research Unit, School of Medicine, Université de la Méditerranée, 27 bd Jean Moulin, Marseille cedex 05, F-13385, France; 2 Departments of Neurology and CRMBM CNRS6612, Timone University Hospital, APHM, Marseille, France; 3 Department of Mathematics, Faculté des Sciences de Luminy, Aix-Marseille University, Marseille, France

**Keywords:** Multiple sclerosis, Quality of life, Response shift, Random forest, MusiQoL, SF-36, Longitudinal studies, Variable importance

## Abstract

**Background:**

Multiple sclerosis (MS), a common neurodegenerative disease, has well-described associations with quality of life (QoL) impairment. QoL changes found in longitudinal studies are difficult to interpret due to the potential response shift (RS) corresponding to respondents’ changing standards, values, and conceptualization of QoL. This study proposes to test the capacity of Random Forest (RF) for detecting RS reprioritization as the relative importance of QoL domains’ changes over time.

**Methods:**

This was a longitudinal observational study. The main inclusion criteria were patients 18 years old or more with relapsing-remitting multiple sclerosis. Every 6 months up to month 24, QoL was recorded using generic and MS-specific questionnaires (MusiQoL and SF-36). At 24 months, individuals were divided into two ‘disability change’ groups: worsened and not-worsened patients. The RF method was performed based on Breiman’s description. Analyses were performed to determine which QoL scores of SF-36 predicted the MusiQoL index. The average variable importance (AVI) was estimated.

**Results:**

A total of 417 (79.6%) patients were defined as not-worsened and 107 (20.4%) as worsened. A clear RS was identified in worsened patients. While the mental score AVI was almost one third higher than the physical score AVI at 12 months, it was 1.5 times lower at 24 months.

**Conclusion:**

This work confirms that the RF method offers a useful statistical approach for RS detection. How to integrate the RS in the interpretation of QoL scores remains a challenge for future research.

**Trial registration:**

ClinicalTrials.gov identifier: NCT00702065

## Background

Regulatory agencies such as the Food and Drug Administration in the United States, the National Institute for Health and Clinical Excellence in England, and the National Authority for Health in France recommend assessing the quality of life (QoL) in patients with chronic disease. Particularly in the field of multiple sclerosis (MS), QoL is recognized as a major outcome measure for assessing health, evaluating treatment, and managing care [1,2]. MS, the most common neurodegenerative disease in young adults, has well-described associations with QoL impairment [3,4].

QoL is a subjective measure of a patient’s life satisfaction that is affected by many factors related to patients’ intrinsic characteristics, such as mood, coping mechanisms, disease state/progression, and factors related to environmental characteristics, such as life experiences and emotional support. Evaluations of change in QoL are important for tracking the progression of the impact of the disease. QoL changes found in longitudinal studies are difficult to interpret. Are these changes due to a true change of the QoL level or to respondents’ changing standards, values, or conceptualizations [5,6]? This phenomenon is also well described and is referred to as a ‘response shift’ (RS). Classically, three types of RS have been distinguished: (a) changes in internal standards of measurement (recalibration), (b) changes in the priority (i.e., importance) of the component domains of the target construct (reprioritization), and (c) redefinition of the target construct (reconceptualization).

Several statistical methods have been proposed to detect an RS [5], specifically in MS populations: the then-test, structural equation modeling (SEM) [7], latent trajectory analysis of residuals [8], and more recently, recursive partitioning tree analysis as a data mining method [9]. Each method has its own specific advantages and limitations that have been clearly discussed [10]. It would be premature to conclude which method is best for detecting an RS. The variety of methods developed illustrates the complexity and difficulty in detecting and measuring an RS.

The Random Forest (RF) method developed by Breiman [11,12] is mainly used as a predictive approach. It has become a popular technique because the RF classification and regression models are versatile. The RF method has high prediction accuracy compared to other classification and regression algorithms [13]. There are numerous examples of the application of the RF in a variety of fields [14], specifically in genomics research [15] and genetic association studies [16]. The method provides an original variable’s importance index for classification and regression that can be applied in other fields [14]—for example, to assess the RS in the reprioritization component of QoL assessments.

This study proposes to test the capacity of the RF approach for detecting RS reprioritization as the relative importance of QoL domains change over time.

The manuscript is organized as follows:

- the methods section, including the study design and setting, a brief description of RF specifications and how we use the RF method to detect RS,

- the results section containing the main findings of the analysis,

- and the discussion section, including the strengths and limitations of the RF method and opportunities for further research.

## Methods

### Study design and setting

This was a multicenter, multiregional, longitudinal observational study carried out at 32 centers in 12 countries: Argentina, Australia, Austria, Germany, Spain, France, Israel, Italy, Norway, Turkey, the United Kingdom, and the United States [17] (Additional file 1: Table S1).

The inclusion criteria were as follows: patients 18 years old or more with relapsing-remitting multiple sclerosis (RR-MS) according to the McDonald criteria [18,19] with an Expanded Disability Status Scale (EDSS) score lower than 7.0, with or without treatment, followed up as per the local standard of care practices and with a signed informed consent form. Patients suffering from dementia were excluded. All therapeutic decisions during the study were made at the discretion of the treating physician.

### Ethics committee and regulatory requirements

This study (ClinicalTrials.gov identifier: NCT00702065) was performed in accordance with the Declaration of Helsinki and all applicable regulatory authority requirements and national laws (Institutional Review Board or Independent Ethics Committee in accordance with the local requirements of each of the 12 countries). Written informed consent from patients was obtained prior to any study procedures.

### Evaluation times and data collection

The follow-up measurements took place over 24 months after inclusion. At baseline, sociodemographic (age at inclusion, gender, education level, marital status, employment status) and clinical (disease duration) data were obtained. Neurological disability status was assessed using a neurologist-rated EDSS score [20]. QoL was determined using the MusiQoL and SF-36 questionnaires when patients attended their local neurological clinic. The MusiQoL questionnaire is a self-administered, multi-dimensional, patient-based QoL instrument comprising 31 items that describe nine dimensions (activity of daily living, psychological well-being, relationships with friends, symptoms, relationships with family, relationship with the healthcare system, sentimental and sexual life, coping, and rejection) [21]. MusiQoL provides a global index score, which is calculated as the mean of the individual dimension scores. The SF-36 is composed of 36 items that are used to calculate the following eight scale scores: physical functioning (PF), social functioning (SF), role–physical (RP), role–emotional (RE), mental health (MH), vitality (Vi), bodily pain (BP), and general health (GH) [22]. Two composite summary measures are also calculated: the Physical Component Summary (PCS) and the Mental Component Summary (MCS) scores. The PCS and MCS scores are norm-based, using a linear T-score transformation with a mean (standard deviation [SD]) of 50 [10]). Both the MusiQoL and SF-36 yield scores on a 0–100 scale, in which 0 represents the lowest and 100 the highest QoL.

Every 6 months up to month 24, the EDSS and QoL were recorded: at baseline (M0), 6 months (M6), 12 months (M12), 18 months (M18), and 24 months post-inclusion (M24).

### Definition of disability deterioration

At 24 months, individuals were divided into two ‘disability change’ groups according to the following neurological standards [23,24]: 1. worsened patients experienced clinically meaningful worsening in the EDSS is defined as an increase of one point if the EDSS was less than 5.5, or by half a point if the EDSS was between 5.5 and 7.0, between the baseline and 24-month EDSS scores; 2. not-worsened patients comprised all other cases.

The not-worsened group was used as a control group in the analysis under the assumption that they were not prone to response shifts in perceived QoL.

### Data analysis

#### Classification and regression trees

The Classification and Regression Trees (CART) method [25] is a binary splitting method that recursively partitions the data set into disjoint subgroups, called the leafs. It uses two algorithms. The first algorithm iteratively splits the data set into two sub-samples according to a binary rule such as “PCS < 50”. The splitting rule is based on one of the explanatory variables and on a threshold for this variable. It is chosen in such a way as to minimize the heterogeneity of the obtained subsamples for a continuous outcome. Regression trees are constructed using the “deviance” criterion.

The two obtained sub-samples are then recursively partitioned in the same way until there are too few observations (usually five) in the obtained samples (other stopping rules are available). This procedure yields a tree that may have too many terminal nodes. The mean value of the output variable is assigned to each leaf, computed over the observations within the corresponding region.

To avoid overfitting the data when using this tree, a pruning algorithm is used to select an optimal sub-tree.

#### The random forest method

Random Forests [11] is an ensemble method that aggregates K trees similar to the ones constructed with CART, each one grown using a bootstrap sample of the original data set. Each tree in the forest uses only a subset of the explanatory variables at each node. The trees are not pruned. The prediction given by an RF is the mean of the predictions given by the K trees in the forest when using regression trees.

#### Variable importance

As the trees in the forest are developed using bootstrap samples of the original data set, the Out-of-Bag (OOB) samples are used as test samples. The performance of each tree is computed over the corresponding OOB sample. The observations of each variable in the OOB sample are randomly permuted, and the trees’ performance is computed over the perturbed OOB samples. A variable's importance (VI) is defined as the mean relative decrease in the trees’ performance when the observations of this variable in the OOB sample are randomly permuted. To obtain more stable assessments of each VI, we run the RF K=300 times and use the average VI over the K runs.

#### Detecting response shift reprioritization with random forest

We investigated the importance of different explanatory variables in the global MusiQoL index forecast. To do this, we calculated the VI by the RF method based on two models.

$$M^{\left(1\right)}{\text{Global}}_{\text{Index}}=f\left(\text{PCS},\text{MCS},X\right)$$

$$M^{\left(2\right)}{\text{Global}}_{\text{Index}}=f\left(\text{PF},\text{RP},\text{VI},\text{BP},\text{SF},\text{RE},\text{MH},\text{GH},X\right)$$

Where

$$\begin{array}{l} X=(\text{Age},\text{Gender},\text{Education}\;\text{Level},\text{Marital}\;\text{Status},\\ \;\text{Employment}\;\text{Status},\text{Disease}\;\text{Duration}). \end{array}$$

Model *M*^*(2)*^ is more refined than *M*^*(1)*^. We adjusted these two models separately for the worsened group and the not-worsened group at each moment t=0,…,4. In this way, we obtained the average of VI (AVI) that evolved with time for each explanatory variable $\tilde{X}\;\text{AV}I_{t}\left(\tilde{X}\right)$. We compared the evolution of AVI for each variable in the two groups. Crossing curves were considered an effect of reprioritization.

To control the difference in baseline EDSS scores between the worsened and not-worsened groups, supplementary analyses were performed on baseline EDSS score-matched groups (100 worsened patients and 100 not-worsened patients).

## Results

### Sample characteristics

The sample included 580 patients enrolled from 12 countries between November 2007 and October 2010. The 24-month EDSS was available for 524 of 536 patients. A total of 417 (79.6%) patients were defined as not-worsened and 107 (20.4%) patients were defined as worsened. Table 1 shows the baseline demographic and clinical characteristics of the worsened and not-worsened subjects.

**Table 1:** Baseline sociodemographic and clinical patient characteristics

		**Total sample°** **N=580**	Worsened patients* N=107	Not-worsened patients*	**p**
Female, n (%)		419 (72.2)	75 (70.1)	300 (71.9)	0.71
Age (years)	M (SD)	41.3 (10.2)	43.2 (10.2)	40.9 (10.1)	**0.03**
	Min, max	18, 71	19, 64	18, 69	
Marital status, n (%)	Cohabiting/married	393 (67.8)	73 (68.2)	282 (67.6)	0.91
	Divorced/separated/single/widowed	187 (32.2)	34 (31.8)	73 (68.2)	
Employment status, n (%)	Employed	335 (57.8)	59 (55.1)	241 (57.8)	0.62
	Unemployed/homemaker/retired/student	245 (42.2)	48 (44.9)	176 (42.2)	
Educational level, n (%)	Elementary school	113 (19.5)	31 (28.4)	76 (18.2)	**0.03**
	College	81 (14.0)	9 (8.3)	61 (14.6)	
	High school/university	386 (66.6)	67 (63.3)	280 (67.1)	
EDSS score	M (SD)	2.9 (1.9)	3.5 (2.2)	2.8 (1.8)	**<10**^**-3**^
	Median	2.5	4.0	2.0	
	Min, max	0.0, 7.5	0.0, 7.0	0.0, 7.0	
MS course, n (%)	Relapsing-remitting	510 (87.9)	79 (73.8)	381 (91.4)	**<10**^**-3**^
	Secondary progressive	53 (9.1)	18 (16.8)	30 (7.2)	
	Primary progressive	12 (2.1)	8 (7.5)	2 (0.5)	
	Primary relapsing	5 (0.9)	2 (1.9)	4 (1.0)	
Time since first MS symptom (years)	M (SD)	10.0 (7.5)	10.2 (8.1)	9.9 (7.2)	0.73
	Min, max	0, 45	0, 45	0.0, 40	

M (SD), mean (standard deviation);

EDSS, Expanded Disability Status Scale; MS, multiple sclerosis;

° the 24-month EDSS was not available for 56 individuals.

* defined as worsened or not-worsened based on change in EDSS score from baseline to month 24 according to the definition of Lublin [Lublin 1996] and Kappos [Kappos 2004];

Bold values: p-value < 0.05.

### Response shift detection on MusiQoL index

**Figure 1:**
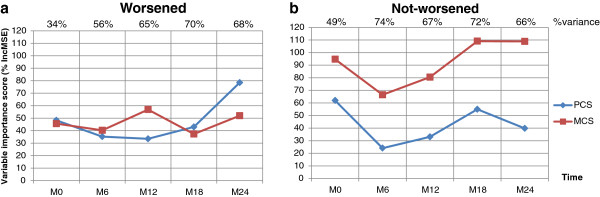
**Average of variable importance of mental and physical composite scores of SF-36 to MusiQoL index prediction.** Figure **1a**. Worsened individuals (n=107). Figure **1b**. Not-worsened individuals (n=417)

**Figure 2:**
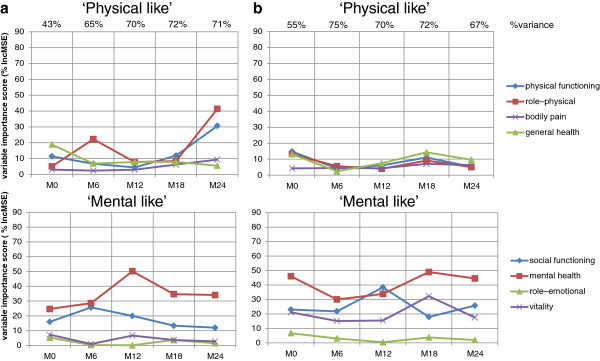
**Average of variable importance of dimensions of SF-36 to MusiQoL index prediction.** Figure **2a**. Worsened individuals (n=107). Figure **2b**. Not-worsened individuals (n=417)

The results are provided in Figure 1 and Figure 2. The proportion of total variance was higher than 55% for each global index model using MCS and PCS variables from M6 to M24, both for worsened and not-worsened individuals (at 24 months, 68% and 66%, respectively). Figure 1a identifies a clear RS in the worsened patients based on the crossing of the MCS and PCS curves over time. In the patients, the MCS and PCS AVI were close at M0, and the MCS AVI was almost one-third higher than the PCS AVI at M12. However, the AVI of PCS was 1.5 times greater than the AVI of MCS at M24. In the worsened patients, the reprioritization RS related specifically to the ‘physical-like’ dimensions of the SF-36 RP and PF dimensions and not the ‘mental-like’ dimensions (Figure 2a). Figure 1b shows the absence of an RS in not-worsened patients with the curves that did not cross over time and with the MCS and PCS AVI progressing symmetrically. In the not-worsened patients, the order of AVI of the SF-36 dimensions (Figure 2b) did not obviously differ between M0 and M24. At M24, the proportion of total variance for the models using the SF-36 dimensions accounted for 71 and 67% for the worsened and not-worsened groups, respectively.

The results of the baseline EDSS-matched groups are detailed in additional figures (Additional file 2: Figure S1 and Additional file 3: Figure S2). The findings were globally similar. One discrepancy concerns the not-worsened patients. While the MCS and PCS AVI progressed symmetrically in the entire sample, the 2 curves were close at M12 in the matched groups.

## Discussion

In longitudinal studies, the fundamental assumption is that the measures are interpretable across time; however, when an RS occurs, this assumption is invalidated because an RS makes change difficult to assess. It is not uncommon for MS patients to report improved mental health status despite severe impairments in physical functioning [9]. When an RS is present, conventional statistical analyses might not detect true change in the measures [26,27]. It is critical for researchers and clinicians to have access to methods for detecting the presence of RS in their data. While several methods were previously used for this purpose, to our knowledge, this is the first study that assesses RS detection using the RF method. The RF method identified patterns of an RS in a global QoL change score. The reprioritization aspect of the RS was recognized through the qualitative differences of the importance of QoL specific domains that were retained by RF analysis.

Using the RF method, the RS was well identified in our worsened population. In this group, we observed that the mental composite score became more important during the twelve months following inclusion, while the importance of mental and physical aspects was close at the initial evaluation. This reprioritization effect should reflect a reaction of psychological compensation highlighted by the specific increase of the importance of the mental health dimension over time. The natural evolution of the disease generally includes deterioration and disability. During the second year of follow-up, the order of prioritization was inverted, with the greatest importance given to the physical component. Among the ‘physical-like’ dimensions of the SF-36, we observed a greater importance of physical functioning and role physical dimensions compared to both bodily pain and general health dimensions, for which the scores were relatively stable over time. This finding can be explained by the fact that the disease is not particularly painful and does not affect general health in the short term.

In the not-worsened population, no crossing of the curves was observed during the 24-month follow-up. The mental composite scores had a greater and, consequently, more important impact on the global quality of life index compared to the physical composite scores from the initial evaluation. In this population, the specific analysis of the ‘mental-like’ dimensions indicated that social functioning was clearly an important dimension, showing higher importance indices than the vitality and role emotional dimensions. In contrast, in the worsened population, the three dimensions showed similar importance, reflecting a lower priority for the social domain of the QoL domain. In our study, the lower importance of social life in this group is independent of marital status, although a relationship between the two parameters was previously reported elsewhere, specifically in MS [28]. Considering marital status as an indirect marker of global social interactions, we thus hypothesize that an MS patient with a severe disease course would anticipate a decrease in his/her social interactions. This reaction would result from the patient’s behavior and beliefs related to the disease. On the contrary, the reprioritization phenomenon found for the social dimension in MS individuals presenting with less severe disease may reflect a willingness to adapt to their situation.

Other methods to detect reprioritization RS have already been developed, specifically in MS populations.

The design-based approaches, specifically the then-test approach [5], assess the self-reported patient quality of life at two different times and calculate the difference between the first time (pre-test) and the last time (then-test). Such methods are sensitive and biased and tend to be restricted to retrospective studies [29].

Structural equation modeling (a model-based approach) tests for a change in the magnitude of factor loadings on a common latent variable over time [30,31]. This approach cannot always be implemented in studies with small sample sizes because the larger number of parameters to estimate may result in a lack of model convergence. The order in which parameters are tested can affect the conclusion. If a substantial portion of the sample has not undergone an RS, the method is more likely to conclude that the RS did not occur.

More recently, the RS was tested using a recursive partitioning tree analysis that is based on the disease trajectory [9,32]. A tree is created for each disease trajectory group. The order of the disability domain indicates reprioritization. This relatively recent data mining method shows promise for identifying small changes in patient-reported outcomes scores over time.

The method based on latent trajectory analysis was centered and used to create trajectories [33]. An RS was hypothesized to be present when an individual's centered residuals showed a pattern of fluctuation over time. This method does not determine the type of RS that occurred, but it is used to identify subgroups of the population who present an RS.

Finally, methods based on the item response theory should be tested.

The RF method presents several advantages. First, the combination of several trees in a forest results in a stronger classification predictor compared to a single tree. Cross-validation procedures to assess the classification performance of the model are unnecessary because they are already built in, as each tree in the forest has its own training and test (OOB) data. Third, RF are non-parametric, non-linear stable models; no assumptions about the form of underlying relationships between the predictor variables and the response are made [34]. Fourth, variable importance may be assessed. Finally, the RF algorithm is available in many different open source software packages. Our choice of the RandomForest package [35], available as an R implementation of the original RF code [36], relied on its wide distribution, ease of use, and the benefit from R data processing functionalities.

The main drawback of our approach is that it only detects the reprioritization component of RS. The role of reprioritization in the score is not quantified. The random forest variable importance measures may be biased in situations where potential predictor variables vary in their scale of measurement or their number of categories [10]. The method does not provide a statistical test for evaluating the assumption of differences between two importance variable scores, making it difficult to give a clear interpretation when the importance measures are close. A test comparing the score curves should provide an objective decision tool for this purpose.

### Strengths and limitations

This study has several strengths and limitations.

The RS phenomenon should not be restricted to RS detection. Future research should be developed to address the remaining essential question: Does the RS need to be integrated into the interpretation of QoL score changes, and how can the weight of the RS in the QoL measure be determined when an RS is detected? The need to restore the usefulness and credibility of the QoL assessment has been recently discussed [37,38]; answering this question will contribute to the reintegration of QoL data into clinical practice.

The nature of the use of the QoL questionnaire should be investigated. Some authors expected that disease-specific measures would be less susceptible to a response shift because they query specific symptoms or functional limitations more than generic measures [39]. We do not accept this assumption because we do not consider MusiQoL to be a symptom-function measure. MusiQoL is a well-validated multidimensional instrument assessing physical, mental, and social domains. Nevertheless, our analyses were performed on an index that is not expected to provide the most sensitive score of changes in the MusiQoL [21]. This restriction illustrates the results more clearly. Future works should provide data from MusiQoL dimension scores that more accurately demonstrate the RS.

Our study investigated the RS phenomena in the global MusiQoL index. It would be of interest to analyze the RS in the SF36 scores in order to make comparisons of the RS among different diseases.

Another important aspect of this study concerns the appraisal process of the RS, which is not directly measured in the present work. In the absence of an external criterion for the RS (pleasure appraisal processes), an RS interpretation of results will remain disputable [10,40]. Future research should measure the RS with direct measures of appraisal.

Future explorations should be performed to compare the capacity of the RF method for detecting the RS with other usual methods and of the degree of convergence of the isolated phenomena.

## Conclusion

Investigation of the response shift in multiple sclerosis is required to establish a strong construct. This work suggests that the random forest method offers a useful statistical approach to response shift detection.

## Abbreviations

AVI: Average variable importance; CART: Classification and Regression Trees; EDSS: Expanded Disability Status Scale; MusiQoL: Multiple Sclerosis International Quality of Life; MS: Multiple sclerosis; OOB: Out-of-Bag; QoL: Quality of life; RF: Random Forest; RR-MS: Relapsing-remitting multiple sclerosis; RS: Response shift; SEM: Structural equation modeling; SF36: Short Form 36; VI: Variable’s importance; ADL: Activity of daily living; PWB: Psychological well-being; RFr: Relationships with friends; SPT: Symptoms; RFa: Relationships with family; RHCS: Relationships with health care system; SSL: Sentimental and sexual life; COP: Coping; REJ: Rejection; PF: Physical function; SF: Social function; RP: Role physical; RE: Role emotional; MH: Mental health; Vi: Vitality; BP: Bodily pain; GH: General health; PCS: Physical composite score; MCS: Mental composite score.

## Competing interest

The authors declare that they have no competing interests.

## Authors’ contributions

Conception and design: MB, BG, PA. Study coordination: JP, BG, PA. Inclusion and clinical data collection: JP. Analysis of data: MB, AL, KB. Interpretation of data: MB, AL, KB, PMF, JP, PA. Drafting and writing of manuscript: MB, KB, BG, PA. Revision of manuscript: MB, AL, KB, PMF, JP, BG, PA. All authors read and approved the final manuscript.

## Pre-publication history

The pre-publication history for this paper can be accessed here:

http://www.biomedcentral.com/1471-2288/13/20/prepub

## Supplementary Material

Additional file 1: Table S1 Investigators and centers. Click here for file

Additional file 2: Figure S1 Average of variable importance of mental and physical composite scores of SF-36 to MusiQoL index prediction on baseline EDSS score matched groups. Additional Figure 1a. Worsened individuals (n=100). Additional Figure 1b. Not-worsened individuals (n=100). Click here for file

Additional file 3: Figure S2 Average of variable importance of dimensions of SF-36 to MusiQoL index prediction on baseline EDSS score matched groups. Additional Figure 2a. Worsened individuals (n=100). Additional Figure 2b. Not-worsened individuals (n=100). Click here for file
