# Supplementary material for: Using the random forest method to detect a response shift in the quality of life of multiple sclerosis patients: a cohort study
Source: BMC Med Res Methodol. 2013 Feb 15;13:20. doi: 10.1186/1471-2288-13-20 (PMC3626785; doi:10.1186/1471-2288-13-20)
Supplement: Additional file 1: Table S1 — Investigators and centers. [file 1471-2288-13-20-S1.doc]

**Additional file 1: table S**1. Investigators and centers

| 12 countries | 32 sites | Prof. / Dr |
| --- | --- | --- |
| Argentina | Hospital Italiano, Buenos Aires  FLENI, Raúl Carrea Institute for Neurological Research, Buenos Aires  Intituto de Neurociencias, Buenos Aires | Patrucco  Fiol  Caceres |
| Australia | The Royal Melbourne Hospital, Melbourne  Eastern Health MS Clinical and Research Unit, Box Hill Hospital, Victoria  Clinical Neurosciences, St Vincent Hospital, Melbourne  Royal Prince Alfred Hospital, Sydney  John Hunter Hospital, Newcastle | King  Butzkueven  Paine  Barnett  Lechner Scott |
| Austria | Barmherzigen Hospital, St Veit ad Glan  Department of Neurology, Kaiser Franz Josef Hospital, Vienna  Allgemeines Hospital, Vienna | Diez  Urbanits  Ransmayer |
| France | St Philibert Hospital, Lomme  Department of Neurology, Timone University Hospital, Marseille | Hautecoeur  Pelletier |
| Germany | Neurologic Clinic, St. Josef Hospital, Bochum  Neurologic Clinic, Technischen University, Munich  Neurological Rehabilitation Center Quellenhof, Bad Wildbad | Chan  Hemmer  Flachenecker |
| Israel | Bnei-Zion Medical Center, Haifa  Edith Wolfson Medical Center, Holon  Rambam Medical Center, Haifa  Haemek Medical Center, Afula  Barzilai Medical Center, Ashkelon | Weller  Gilad  Shifrin  Bloch  Milo |
| Italy | Institute of Neurological Sciences IRCSS AUSL, Bologna  Neurologica Hospital, Modena  Carregi hospital, University of Florence, Florence  Azienda Ospedaliera Ospedali Riuniti Villa Sofia-Cervello, Palermo  A Perrino Hospital, Brindisi | Sergio Stecchi  Patrizia Sola  Maria Pia Amato  Salvatore Cottone  Bruno Passarella |
| Norway | Akershus University Hospital, Lørenskog, | Antonie Giaever Beiske |
| Spain | Hospital Regional Universitario Carlos Haya, Málaga | Oscar Fernandez |
| Turkey | Dokuz Eylül University, Izmir | Idiman |
| UK | Queen's Medical Centre, Nottingham  Southern General Hospital, Glasgow | Cris Constantinescu  Colin O’Leary |
| US | Banner Good Samaritan Hospital, Phoenix | Barry A. Hendin |
