# Supplementary material for: Using the random forest method to detect a response shift in the quality of life of multiple sclerosis patients: a cohort study
Source: BMC Med Res Methodol. 2013 Feb 15;13:20. doi: 10.1186/1471-2288-13-20 (PMC3626785; doi:10.1186/1471-2288-13-20)
Supplement: Additional file 2: Figure S1 — Average of variable importance of mental and physical composite scores of SF-36 to MusiQoL index prediction on baseline EDSS score matched groups. Additional Figure 1a. Worsened individuals (n=100). Additional Figure 1b. Not-worsened individuals (n=100). [file 1471-2288-13-20-S2.pptx]

## Slide 1
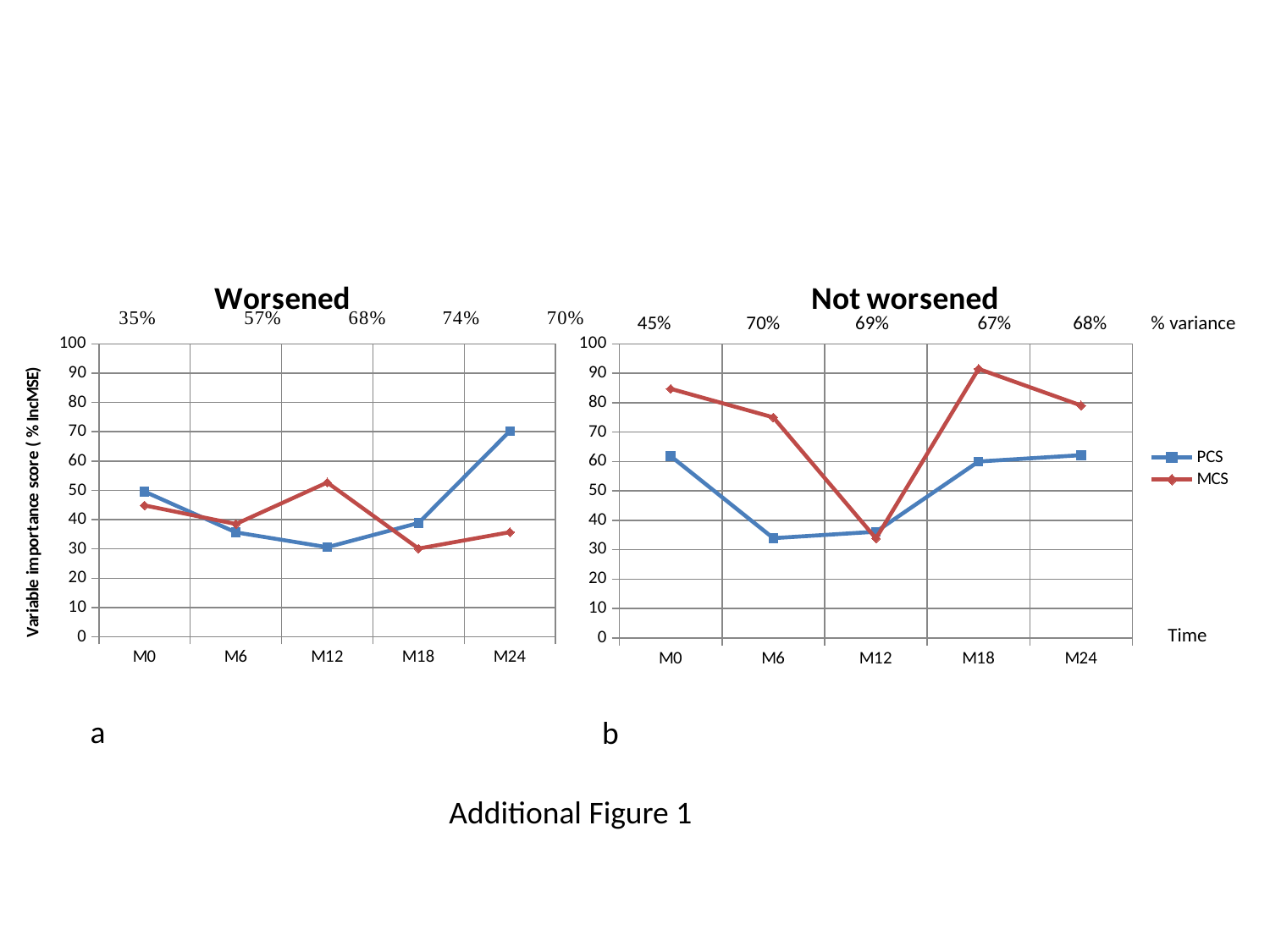

### Chart: Worsened
| Category | PCS | MCS |
|---|---|---|
| M0 | 49.57101 | 44.85544 |
| M6 | 35.6521 | 38.50512 |
| M12 | 30.59194 | 52.66559 |
| M18 | 38.8631 | 30.12677 |
| M24 | 70.22602 | 35.7247 |
### Chart: Not worsened
| Category | PCS | MCS |
|---|---|---|
| M0 | 61.84417 | 84.73381 |
| M6 | 33.90964 | 75.01879 |
| M12 | 36.1041 | 33.83092 |
| M18 | 60.00786 | 91.51284 |
| M24 | 62.16427 | 79.05581 |45% 70% 69% 67% 68% % variance
Time
a
b
Additional Figure 1
