# Supplementary material for: Using the random forest method to detect a response shift in the quality of life of multiple sclerosis patients: a cohort study
Source: BMC Med Res Methodol. 2013 Feb 15;13:20. doi: 10.1186/1471-2288-13-20 (PMC3626785; doi:10.1186/1471-2288-13-20)
Supplement: Additional file 3: Figure S2 — Average of variable importance of dimensions of SF-36 to MusiQoL index prediction on baseline EDSS score matched groups. Additional Figure 2a. Worsened individuals (n=100). Additional Figure 2b. Not-worsened individuals (n=100). [file 1471-2288-13-20-S3.pptx]

## Slide 1
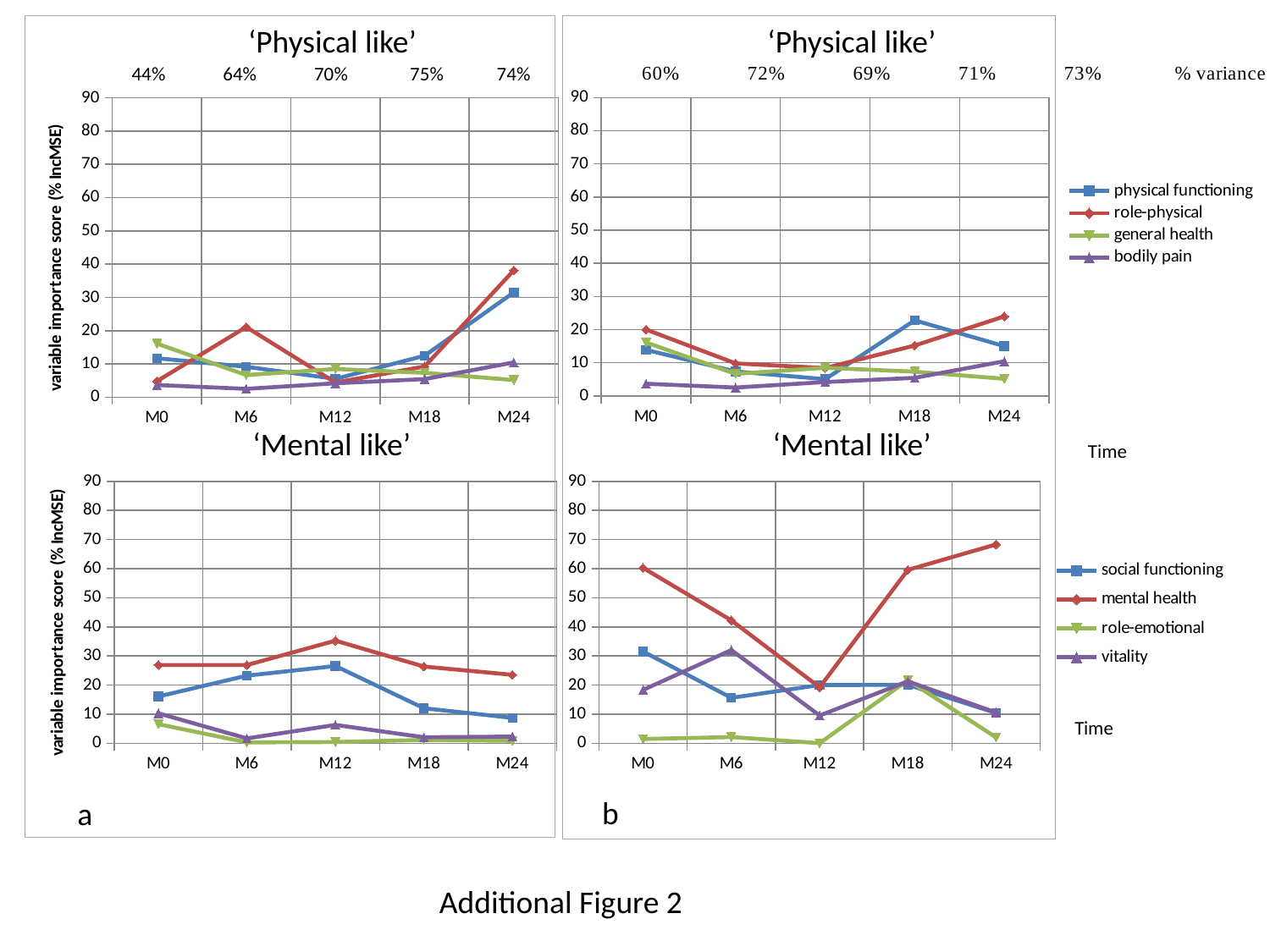

### Chart:
| Category | physical functioning | role-physical | general health | bodily pain |
|---|---|---|---|---|
| M0 | 11.724906 | 4.810222 | 16.105784 | 3.673635 |
| M6 | 9.151914 | 21.096091 | 6.658705 | 2.519943 |
| M12 | 5.521685 | 4.41361 | 8.47539 | 4.183802 |
| M18 | 12.470546 | 9.221975 | 7.315115 | 5.450715 |
| M24 | 31.516462 | 38.142045 | 5.164109 | 10.457694 |
### Chart:
| Category | physical functioning | role-physical | general health | bodily pain |
|---|---|---|---|---|
| M0 | 13.894815 | 20.025224 | 16.105784 | 3.673635 |
| M6 | 7.359981 | 9.799212 | 6.658705 | 2.519943 |
| M12 | 5.037314 | 8.403408 | 8.47539 | 4.183802 |
| M18 | 22.795608 | 15.203417 | 7.315115 | 5.450715 |
| M24 | 15.009252 | 23.976888 | 5.164109 | 10.457694 |
‘Physical like’
‘Physical like’
 44% 64% 70% 75% 74%
### Chart:
| Category | social functioning | mental health | role-emotional | vitality |
|---|---|---|---|---|
| M0 | 16.083167 | 26.90256 | 6.5894273 | 10.308782 |
| M6 | 23.239183 | 26.88479 | 0.3138211 | 1.690211 |
| M12 | 26.555742 | 35.26707 | 0.4475501 | 6.331288 |
| M18 | 12.065028 | 26.37461 | 1.1672234 | 2.079843 |
| M24 | 8.737811 | 23.54627 | 0.8500166 | 2.323358 |
### Chart:
| Category | social functioning | mental health | role-emotional | vitality |
|---|---|---|---|---|
| M0 | 31.54381 | 60.27186 | 1.4784579 | 18.353804 |
| M6 | 15.63138 | 42.22001 | 2.15504004 | 32.027494 |
| M12 | 20.01771 | 19.25322 | 0.06176494 | 9.542399 |
| M18 | 20.0968 | 59.58357 | 21.71424082 | 21.294952 |
| M24 | 10.4247 | 68.32904 | 2.03089253 | 10.556789 |‘Mental like’
‘Mental like’
Time
b
a
Additional Figure 2
